# Characterization of the clinical and genetic spectrum of autoimmune polyendocrine syndrome type 1 in Chinese case series

**DOI:** 10.1186/s13023-021-01933-y

**Published:** 2021-07-03

**Authors:** Ya-Bing Wang, Ou Wang, Min Nie, Yan Jiang, Mei Li, Wei-Bo Xia, Xiao-Ping Xing

**Affiliations:** 1 Department of Endocrinology, Key Laboratory of Endocrinology of the Ministry of Health, Peking Union Medical College Hospital, Chinese Academy of Medical Science & Peking Union Medical College, Dongcheng District, Shuaifuyuan No.1, Beijing, 100730 China; 2 Department of Endocrinology, Beijing Friendship Hospital, Capital Medical University, Beijing, 100730 China

**Keywords:** Autoimmune polyendocrine syndrome type 1, Hypoparathyroidism, *AIRE* gene, Chinese

## Abstract

**Background:**

Autoimmune polyendocrine syndrome type 1 (APS1) is a hereditary disease caused by mutations in the *AIRE* gene with both endocrine and non-endocrine organ involvement. The existing data from China are limited, and this study aims to describe the phenotypes and genetic characterization in Chinese APS1 patients. In this single-center, retrospective, observational study, comprehensive endocrine and extra-endocrine manifestations were collected, and genetic analysis in *AIRE* was conducted in patients with APS1 between the years of 1984 and 2018 at Peking Union Medical College Hospital.

**Results:**

In total, 13 patients from 12 unrelated families were enrolled, seven of whom were female, with hypoparathyroidism, chronic mucocutaneous candidiasis, and Addison’s disease being the most frequently observed manifestations. Up to 84.7% presented with two or three of the above-mentioned manifestations, and nearly 4.9 ± 1.8 components presented in patients aged 21.2 ± 7.9 years old. Several less common phenotypes, such as myeloproliferative disease, pure red cell aplasia, renal tubular acidosis, asplenia, autoimmune hepatitis, and ankylosing spondylitis, were also observed in patients. Altogether, seven different *AIRE* mutations were found in six patients, four of which (K161fs, G208V, A246fs, and L308F) had not been previously reported in patients with APS1.

**Conclusion:**

We have provided a comprehensive profile of Chinese patients with APS1, with less commonly observed features being observed in addition to more regularly seen manifestations. Additionally, different *AIRE* mutations that were observed have expanded the genetic spectrum, which will help with future understanding of the molecular pathogenesis of APS1.

**Supplementary Information:**

The online version contains supplementary material available at 10.1186/s13023-021-01933-y.

## Background

Autoimmune polyendocrine syndrome type 1 (APS1; OMIM #240300) is a rare disease with a prevalence of 1:9000 to 1:25000 in different countries, primarily concentrated within isolated populations [1]. It has traditionally been thought that APS1 is an autosomal recessive genetic disorder caused by mutations in the autoimmune regulator (*AIRE*) gene. Under normal conditions, naive autoreactive T cells in the thymus are regulated by *AIRE*, defects of which may cause tissue-specific autoimmunity through defective elimination of autoreactive T cells [1]. Thus, patients with APS1 may develop multiple manifestations, and they mainly present with a classic triad of chronic mucocutaneous candidiasis (CMC), Addison’s disease (AD), and hypoparathyroidism (HP), as well as other less common manifestations such as enamel hypoplasia, alopecia, type 1 diabetes, and autoimmune disease, etc. [2]. More than 100 *AIRE* mutations have been linked to APS1, most of which are of autosomal recessive inheritance. However, some of them follow a dominant inheritance pattern, such as those located in the SAND domain and the PHD1 domain [3, 4]. Previous studies have demonstrated that some genotypes can result in different clinical phenotypes or varying penetrance and severity, with no clear phenotype-genotype correlations having been established.

As a monogenic disease, socioeconomic and environmental factors may also affect the penetrance. Different races may have different profiles [2]. Before the classical diagnostic criteria, where at least two of the three manifestations are present, some patients showed non-classical symptoms for many years [5]. Because of the high variability, incomplete penetrance, and temporal heterogeneity of different manifestations, establishing a diagnosis is often difficult or delayed. So far, the majority of studies on the clinical and genetic profile of APS1 have been reported from European countries, while data from China are scarce and primarily from case reports.

The purpose of the present study was to analyze the clinical features and *AIRE* mutations in a single-center cohort of Chinese APS1 patients. To the best of our knowledge, this is the first case series in China for APS1.

## Results

### Clinical presentation

#### Ascertaining of patients with APS1 presenting the classic triad

A total of 13 patients (seven females and six males) from 12 unrelated families were enrolled in this study. Two sibling patients came from the same family with consanguineous parents. An overview of the disease components is displayed in Table 1. Until the age of 21.2 ± 7.9 years old at the last follow-up, six patients (46.2%) displayed the classic triad and five patients (38.5%) had two manifestations of the triad. Eleven patients were clinically diagnosed and five of them were further confirmed by genetic testing. Another two patients had only one of the triad. One such patient was diagnosed due to carrying a pathogenic *AIRE* mutation, and the other by a family history of APS1.

**Table 1:** The prevalence of APS1 (n = 13) related components

Components	Prevalence (%)
Classic triad	
Hypoparathyroidism	12 (92.3)
Chronic mucocutaneous candidiasis	9 (69.2)
Addison’s disease	9 (69.2)
Any of two	5 (38.5)
All three	6 (46.2)
Other endocrinopathies	
Subclinical hypothyroidism	6 (46.1)
Primary amenorrhea	2 (15.4)
Ectodermal dysplasia	
Enamel dysplasia and nail dystrophy	6 (46.1)
Hair loss	8 (61.5)
Ocular manifestations	
Keratitis	1 (7.7)
Retinitis pigmentation	2 (15.4)
Hematological diseases	
Myeloproliferative disease	1 (7.7)
Pure red cell aplasia	1 (7.7)
Other less common complications	
Intestinal malabsorption	3 (23.1)
Renal tubular acidosis	1 (7.7)
Asplenia	1 (7.7)
Autoimmune hepatitis	1 (7.7)
Ankylosing spondylitis	1 (7.7)

APS1, autoimmune polyendocrine syndrome type 1

**Fig. 1:**
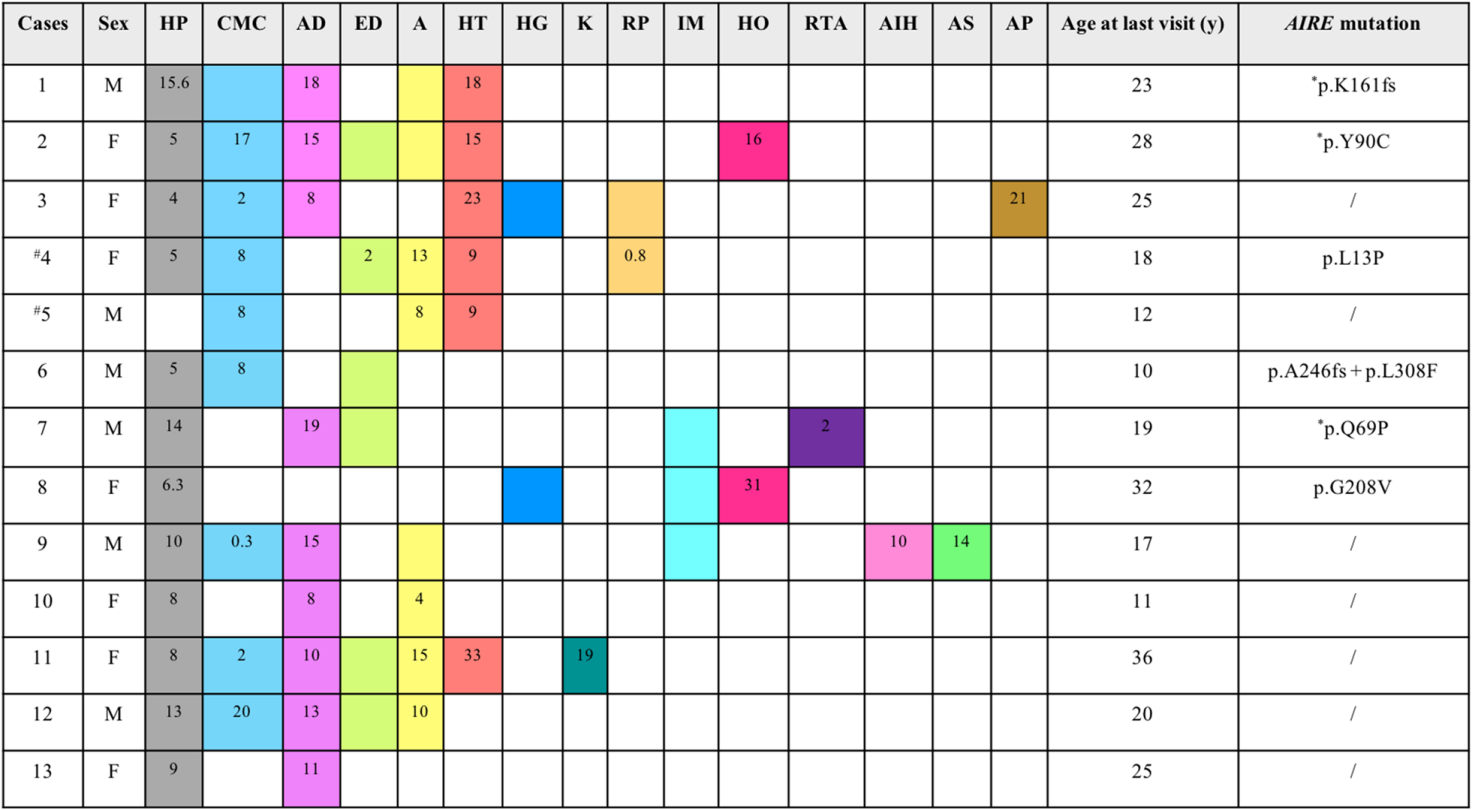
Clinical spectrum and genotype in patient with APS1. APS1, autoimmune polyendocrine syndrome type 1; HP, hypoparathyroidism; CMC, chronic mucocutaneous candidiasis; AD, Addison’s disease; ED, ectodermal dysplasia, including enamel dysplasia and nail dystrophy; A, alopecia; HT, hypothyroidism; HG, hypergonadotropic hypogonadism; K, keratitis; RP, retinitis pigmentosa; IM, intestinal malabsorption; HO, hematopathy; RTA, renal tubular acidosis; AIH, autoimmune hepatitis; AS, ankylosing spondylitis; AP, asplenia; M, male; F, female. ^#^Case 4 and case 5 were siblings, respectively. *Homozygous mutations. The parents of cases 7 and 12 were consanguineous marriages. The onset age (year) of different components known were shown in the pane. GenBank accession number of *AIRE*: NM_000383

Clinical features and genetic findings in each patient are outlined in Fig. 1. All 13 patients presented with an average number of 4.9 ± 1.8 APS1 related features. Age at onset of the first manifestation was 5.5 ± 4.3 years. The initial manifestations were HP in nine patients (69.2%) and CMC in four patients (30.8%). Twelve cases (92.3%) presented with HP, with onset or diagnostic age of 8.6 ± 3.9 years. CMC including oral candidiasis, tinea manus, and tinea pedis occurred in nine cases (69.2%) with the average onset age being 8.2 ± 7.1 years (range, three to four months after birth to 20 years). Nine cases (69.2%) with AD were diagnosed at an average of 13.0 ± 4.1 years.

#### Other endocrinopathies

Six cases (46.1%) developed subclinical hypothyroidism. Two female patients presented with primary amenorrhea. Islet cell antibodies, indicative of type 1 diabetes onset, were detected in one patient with normal blood glucose levels during follow-up.

#### Ectodermal dysplasia

Enamel dysplasia and nail dystrophy were found in six cases. Obvious hair loss was reported in eight cases (61.5%), of which three showed alopecia at an average of 7.3 ± 2.5 years.

#### Ocular manifestations

Case 11 had a history of keratitis and two other two patients had progressively decreasing vision in both eyes since childhood. Of these, case three underwent lens implantation for bilateral cataracts at age 5, and her vision reduced only to the perception of light at age 14. She was diagnosed with binocular retinitis pigmentosa by ophthalmic evaluation at age 23. Case four developed nystagmus after birth and ophthalmic examination at 10 months after birth showed retinitis pigmentation, binocular strabismus, and amblyopia. This resulted in weak vision in her right eye and loss of vision in her left eye at the age of eight.

#### Hematological diseases

Case two was found to have increased levels of platelets (620–720 $\times$ 10^9^/L) during a routine examination at the age of 16. Myeloproliferative disease was diagnosed after a comprehensive examination of bone marrow by a hematologist. This patient was not given any treatment and was on long-term follow-up. Case eight was diagnosed as pure red cell aplasia due to anemia with minimal hemoglobin 50 g/L at 31 years old. Bone marrow smear examination indicated active proliferative ability with a granulocyte to erythrocyte ratio of 68:1. Bone marrow biopsy showed no obvious abnormalities. Due to intermittent increases in peripheral lymphocyte proportion, bone marrow immunophenotype analysis was performed. The proportion of T cells was 96%, and CD4/CD8 was significantly inverted. Successive immunotherapies including cyclosporine, stanozolol, and glucocorticoids were effective.

#### Other rare complications

Three patients were diagnosed with intestinal malabsorption due to recurrent diarrhea. Case seven had a positive intrinsic factor antibody but not anemia. This patient also showed signs of polyuria, polydipsia, periodic paralysis, and rickets when he was about two years old. Examination revealed metabolic acidosis, hypokalemia, proteinuria, increased urinary $\mathrm{\beta }$ 2 microglobulins, and low HCO_3_^−^ excretion rate (< 5%) with no secondary factors identified. Thus, he was diagnosed with type 1 renal tubular acidosis (RTA) and received oral alkaline therapy composed of sodium citrate, potassium citrate, and citric acid to maintain a normal blood pH and normokalemia until four years old. Re-examination at the age of 19 indicated a spontaneous remission of RTA.

Case three presented with increased lymphocyte (5.9–7.8 $\times$ 10^9^/L) and platelet counts (393–445 $\times$ 10^9^/L) at the age of 21 with no hematological diseases found by related examinations. Abdominal ultrasound and CT scan examinations showed asplenia, which may be the cause of abnormal blood counts.

Case nine had slightly elevated liver enzymes and inflammatory indicators and low-titer antinuclear antibodies; the patient tested negative for hepatotropic virus at the age of 10 years old. Liver biopsy pathology suggested inflammatory disease, which indicated autoimmune hepatitis. Meanwhile, this patient presented with progressive pain of the knee, ankle, and back at the age of 14, making him unable to walk. HLA-B27 was positive, and the erythrocyte sedimentation rate was elevated. CT and MRI scans of the hip found that this was in line with the characteristics of ankylosing spondylitis. His symptoms related to ankylosing spondylitis achieved remission after regular infliximab treatment.

### AIRE mutations

*AIRE* gene sequencing was performed in six patients and results are displayed in Table 2. Altogether, seven different *AIRE* mutations were detected, including five missense mutations, one small duplication, and one small deletion mutation, among which four were previously unreported. Three patients (case seven, case two, case one) harbored *AIRE* homozygosity mutations (p.Q69P, p.Y90C, p.K161fs), and one of them was from a consanguineous family. These three variants localize to the CARD and NLS domain of AIRE protein. Case six carried a compound heterozygous mutation (p.A246fs and p.L308F), localized to the SAND and the PHD1 domain. Another two cases (case four and case eight) harbored *AIRE* heterozygous mutations (p.L13P and p.G208V, respectively) in the CARD and SAND region, respectively.

**Table 2:** *AIRE* gene mutations in included patients with APS1

Domain	Cases	Location	Nucleotide alteration	Amino acids change	Heterozygosity	MAF in Esp6500/ 1000g_All/ExAC	Predicted in SIFT/Polyphen2-HVAR/MutationTaster	Reported (PubMed ID)
HSR/CARD	4	Exon 1	c.T38C	p.L13P	Het	–/–/–	B/D/D	28911151
	7	Exon 2	c.A206C	p.Q69P	Hom	–/–/–	D/P/D	28540407
	2	Exon 2	c.A269G	p.Y90C	Hom	–/–/–	D/D/D	9837820
NLS	1	Exon 4	c.484dupC	p.K161fs	Hom	–/-/–	–/–/–	No
SAND	8	Exon 5	c.623G > T	p.G208V	Het	–/–/–	D/D/D	No
	6	Exon 6	c.737delC	p.A246fs	Het	–/–/–	–/–/–	No
PHD1	6	Exon 8	c.C922T	p.L308F	Het	–/–/–	D/D/D	No

APS1, autoimmune polyendocrine syndrome type 1. GenBank accession number of *AIRE*: NM_000383; MAF, minimum allele frequencies; ESP, NHLBI Exome Sequencing Project; 1000 g: 1000 genomes browser; ExAC, Exome Aggregation Consortium; SIFT, sorting intolerant form tolerant; Polyphen2-HVAR, polymorphism phenotyping version 2; HSR, homogeneously staining region; CARD, caspase recruitment domain; NLS, nuclear localization signal; PHD, plant homeodomain; Het, heterozygous; Hom, homozygous; “–” indicates that data are not available; B, benign; D, deleterious; P, probably deleterious. CARD/HSR, amino acids 1–105; SAND, amino acids 181–280; two plant homeodomain (PHD) fingers type zinc fingers (amino acids 296–343 and 434–475); four LXXLL domains that are found on coactivators of nuclear receptors (amino acids 7–11, 63–67, 414–418, and 516–520) and a nuclear localization signal (amino acids 100–189)

## Discussion

The present study provides a detailed clinical characterization of Chinese patients with APS1 from a single center. The genetic spectrum of *AIRE* within some of the patients was also analyzed here. To our knowledge, this is the first case series reported to date in China on APS1.

Compared to the classical clinical findings, our results were generally similar to those reported in Finland, Italy, Norway, Iran (in Iranian Jews), and Russia [6–8], and hypoparathyroidism primarily ranked first (79–96%) [5]. Hypoparathyroidism usually occurs prior to 10 years, with a peak age of four to five years of age [2]. In our study, hypoparathyroidism occurred in 92.3% of the patients between the ages of four to 18 years (mean 8.6 ± 3.9 years) and was the first symptom of the triad in about 70% of patients. Previous studies have identified that APS1 accounts for 2.7%–5.2% in childhood-onset patients with hypoparathyroidism [9, 10]. Therefore, all childhood-onset patients with hypoparathyroidism should be systematically evaluated for evidence of APS1, and long-term follow-up was necessary. Similar to hypoparathyroidism, CMC also exhibits high penetrance (70–100%), including in this cohort, but not found in Iranian Jews (17%). In our patients, about 30% had CMC as the first symptom. Unlike previous reports, CMC occurred at a later age in our patients than those reported in the literature (8.2 ± 7.1 years vs. median three years) and was not always the first manifestation. AD was the second most common endocrinopathy with an onset age of 13.0 ± 4.1 years, which was consistent with previous studies. Ectodermal dysplasia was the next common feature as reported. The results of the present study suggested that autoimmune thyroiditis may be the third most common endocrine disease (46.1%), which was higher than those reported previously (4–20%). Since genotypes are not always available, the manifestation of the triad is crucial for diagnosis. It is worth mentioning that more than 80% of cases developed at least two of the triads at 21.2 ± 7.9 years old in our patients. Combined with previous literature, it is suggested that the triad of APS1 mostly appears before the age of 30, and identification of other diseases with similar manifestations should be considered after the age of 30 [1].

In addition to above mentioned common manifestations, several rare manifestations, which may be related to APS1, were also uncovered by the present study. Hematological disease was diagnosed in two patients, one with myeloproliferative disease characterized by thrombocytosis, and another with pure red cell aplasia (PRCA), which is rarely reported [8]. Previous studies found that T cell-mediated inhibition of erythropoiesis may attribute to the etiology of PRCA in APS1 [11]. In our patient with thrombocytosis, no secondary factors were found. One previous case report had attributed the thrombocytosis combined with APS2 to atrophy of the spleen [12]. Splenic atrophy was also observed in one of our patients, who also showed increased lymphocyte and platelet count. Since bone marrow biopsies showed no abnormality, it was speculated that the blood abnormality was related to splenic atrophy. Renal tubular acidosis has previously been reported in 2/30 of a Finnish cohort of patients with APS1 [13]. One of the two patients had a past history of type 1 RTA that resolved and has a normal renal function in follow-up, while the other case of type 1 RTA is related to tubule-interstitial nephritis. In pediatric patients with Sjögren syndrome and systemic lupus erythematosus, renal tubular acidosis was reported [14, 15]. Therefore, it is speculated that immune factors may be the cause of APS1 related RTA. For the patient in RTA remission, the authors did not specify a treatment strategy in the literature. Therefore, more cases may be needed to observe whether the RTA occurring in APS1 patients can spontaneously relieve. Autoimmune hepatitis displayed in one of our patients has been previously reported in APS1 patients with a prevalence of nearly 10% [2, 8]. Ankylosing spondylitis (AS) reported in one of our patients was not reported in patients with APS1 previously. Its association with APS1 was not clear, and whether human leukocyte antigen plays a role relating APS1 to AS needs further study and exploration. In total, an average of five and up to seven manifestations can occur in patients with APS1 in our study, which is consistent with previous literature [8]. Therefore, patients diagnosed with APS1 at any age should be followed up closely to watch for new symptoms.

To analyze the clinical characteristics of Chinese APS1 patients comprehensively, we conducted literature review and analysis of Chinese APS1 patients with complete data reported in previous literature. The clinical spectrum and genotype of the 13 patients in our center and 12 cases described in the literature from 2000 to 2018 [16–25] were combined and displayed in Additional file 1: Table 1 and Figure 1. The *AIRE* genotype was available in six of the twelve cases reported in the literature. There were 14 females, 10 males, and one with unknown gender. The number of clinical manifestations ranged from 2 to 7 (4.4 ± 1.6) with the last evaluation age of 20.5 ± 7.1 years. In addition to the spectrum shown in our patients, two patients developed type 1 diabetes, of which one also had pernicious anemia at the age of 18, two presented with Japanese encephalitis all at the age of seven, and one exhibited megaloblastic anemia by bone marrow biopsy at 18 years old, but intrinsic factor antibody was not measured [20, 22]. Of concern, patients with APS1 may undergo an adverse outcome. In the reported APS1 cases in China, one patient died of diabetic ketosis at the age of 24 years old [20]. The other suffered repeated adrenal crisis due to irregular medication, caused by economic problems, and died from the concomitant diabetic ketosis due to type 1 diabetes at the age of 24 years old [22]. Previous studies demonstrated that the most common cause of death in patients with APS1 was hypocalcemia or adrenal crisis, acute hepatitis, and oral and esophageal squamous cell carcinoma [5, 8]. A recent Finnish study found that the overall disease mortality was increased significantly [26]. In 27.5% (25/91) of cases, the cause of death was associated with APS1, and the median age at death was 36.7 years. This suggested that APS1-related disease should be closely monitored and treatment should be standardized.

Fourteen *AIRE* mutations were identified in twelve patients of our study combined with those from literature in China (Additional file 1: Table 2), 10 of which were not reported out of China so far. Five patients showed four APS1-causing *AIRE* homozygous mutations, among which two were in the CARD domain (p.Q69P, p.Y90C), and three were in the NLS domain (p.G155S, p.K161fs). Three patients presented with compound heterozygous mutations, one with p.A19T and p.R257X, another with p.A246fs and p.L308F, and the third with full *AIRE* gene deletion in one allele and splice site mutation (IVS11+1G>A) in another allele. Heterozygous mutations were found in four patients. Among them, p.L13P and p.T16R were in the CARD domain, and p.G208W and p.G208V were in the SAND domain. To date, more than one hundred mutations have been reported in patients with APS1. Mutations found in Chinese patients have been rarely previously reported. Some mutations found in this group of Chinese patients have been reported in other ethnic groups, such as p.L13P in the Russian [8], p.Y90C in the British [4], p.T16R at the same site of p.T16M reported in Greek [27], p.R257X in the Polish, Italian and Finnish [28–30], and entire *AIRE* gene deletion in the Dutch [31]. In this group of Chinese patients, only two carried the same p.K161fs mutation, and nearly 36% of mutations have been reported in other ethnicities. With growing understanding of the genetic background of the disease, limited research demonstrated that *AIRE* mutation may display an autosomal-dominant pattern of inheritance, mainly in the PHD and SAND domains. Of concern, a substantial proportion of *AIRE* mono-allelic heterozygous mutations (4/12) in this group of Chinese patients were identified in the CARD and SAND domain, and only one case exhibited non-classical features (only hypoparathyroidism was found from the triad). It was previously believed that patients carrying the dominant-inheritance pattern mutations mostly presented with atypical manifestations of APS1 [4]. However, whether the four monoallelic heterozygous mutations exert a dominant negative effect remains unknown since the presence of intronic variants or large deletions cannot be excluded.

Whether genotype can explain the phenotype of APS1-related disease remains unclear. To date, no clear association between genotype and phenotype of APS1-related diseases has been found in previous studies from different ethnicities. Notably, compound heterozygous mutations of *AIRE* can also cause isolated hypoparathyroidism [32]. *AIRE* mutations found in three isolated hypoparathyroidism patients have also been reported in our previous study [10]. Since the clinical phenotype of APS1 was not fully evaluated due to incomplete follow-up, they were not included in this study. Further studies with larger sample size are needed to explore the association between genotype and phenotype.

The main limitation of the present study is the small sample size. Therefore, the characteristics of Chinese patients with APS1 cannot be elucidated sufficiently. Second, because of our study’s retrospective nature, there may be bias with regard to the proportion of each component. Third, AIRE mutations reported in our center were predicted to be APS1-causing. Unfortunately, there are a lack of relevant functional studies to verify their pathogenicity. In particular, for monoallelic mutations in the CARD domain, it is likely that no other mutations have been found, as there is no indication that the missense mutation in the CARD domain impairs the function of wild-type AIRE. Meanwhile, some variations in deep intronic regions as well as larger fragments of deletions (especially for those with homozygous variants) could not be excluded, which may affect the results of the study.

## Conclusions

To the best of our knowledge, this is the first case series of Chinese patients with APS1 in a single center. Classical features in our patients are similar to those reported in the literature, with the exception of a relatively later onset of CMC in our group. Moreover, instances of rare manifestations were first reported in patients with APS1, and 10 different *AIRE* mutations identified had not been previously reported. Thus, our findings help to expand the clinical and genetic spectrum of APS1.

## Methods

### Patients

A total of 13 patients with APS1 who were diagnosed between the years of 1994 and 2018 at the Metabolic Bone-Disease Clinic in the Department of Endocrinology at Peking Union Medical College Hospital were enrolled in this study, including four cases that were previously reported as case reports [33–35]. APS1 was defined as the presence of any two of the triad (including CMC, AD, and HP), any one of the major triads with a positive family history of APS1, or positive *AIRE* mutations [2, 8]. We excluded patients with mimicking triad of APS1 caused by secondary factors, such as POEMS syndrome (n = 1). All patients received a relevant evaluation by the endocrinologist. This study was conducted according to the principles outlined in the Declaration of Helsinki.

### Clinical data collection

General demographic and clinical manifestations of APS1 (including HP, CMC, AD, enamel hypoplasia, alopecia, primary hypothyroidism, hypergonadotropic hypogonadism, type 1 diabetes mellitus, chronic diarrhea, keratitis, retinitis pigmentosa, meningoencephalitis, etc.), as well as related patient information such as sex, onset age or age at diagnosis, were retrospectively retrieved from medical records. Endocrinopathies were diagnosed by endocrinologists. Enamel hypoplasia was confirmed by defects in enamel after excluding other known dental diseases. Eye diseases including keratitis and retinitis pigmentosa were evaluated by ophthalmologists. Patient histories of recurrent diarrhea, meningoencephalitis, and alopecia were self-reported by patients or by their parents and were confirmed by patients’ corresponding medical histories.

### Genotyping and in silico functional prediction of the AIRE mutations

In this study, six out of 13 patients agreed to undergo targeted next-generation sequencing (T-NGS, Novogene, HiSeq 2500 sequencing system), and the self-designed panel included the *AIRE* gene. Genomic DNA extraction and T-NGS were carried out as previously described [10], with Sanger sequencing was performed to validate the *AIRE* mutations. Primers of the *AIRE* gene were designed by Primer 3 software and the sequences are available upon request. Minimum allele frequencies (MAF) were assessed using the 1000genomes browser (http://browser.1000genomes.org/), Exome Aggregation Consortium (ExAC, http://exac.broadinstitute.org/), and the NHLBI Exome Sequencing Project (ESP, http://evs.gs.washington.edu/EVS/). To predict the pathogenicity of the mutations, Sorting Intolerant From Tolerant (SIFT, http://sift.jcvi.rog/), MutationTaster (http://www.mutationtaster.org/), and Polymorphism Phenotyping version 2 (Polyphen-2, http://genetics.bwh.harvard.edu/pph2/) were used. The Human Gene Mutation Database (HGMD, http://www.hgmd.org) was consulted to verify whether the variants had been previously reported.

### Statistical analysis

SPSS version 16.0 (IBM Corp., Armonk, NY, USA) was used to perform statistical analyses. Normally distributed continuous variables are presented as the mean ± SD, and non-normally distributed variables are presented as the median (25th and 75th percentiles).

## Supplementary Information

**Additional file 1.** **Table 1.** Prevalence of Different Components in Chinese Patients with APS1. Abbreviation: APS1: Autoimmune Polyendocrine Syndrome Type 1. **Table 2.** Mutations of the *AIRE* Gene in Chinese Patients with APS1. Abbreviation: APS1: Autoimmune Polyendocrine Syndrome Type 1. GenBank accession number of *AIRE*: NM_000383. HSR: Homogeneously Staining Region; CARD: Caspase Recruitment Domain; NLS: Nuclear Localization Signal; PHD: Plant Homeodomain; Het: Heterozygous; Hom: Homozygous; “-” indicates that data are not available. CARD/HSR, amino acids 1–105; SAND: amino acids 181–280; two plant homeodomain (PHD) fingers type zinc fingers (amino acids 296–343 and 434–475); four LXXLL domains that are found on coactivators of nuclear receptors (amino acids 7–11, 63–67, 414–418, and 516–520) and a nuclear localization signal (amino acids 100–189). **Figure 1.** Clinical Spectrum and Genotype in Patients with APS1 in China. Abbreviation: APS1: autoimmune polyendocrine syndrome type 1; HP: hypoparathyroidism; CMC: chronic mucocutaneous candidiasis; AD: Addison’s disease; HT: hypothyroidism; HG: hypergonadotropic hypogonadism; T1DM: type 1 diabetes mellitus; ED: ectodermal dysplasia, including enamel dysplasia and nail dystrophy; A: alopecia; K: keratitis; RP: retinitis pigmentosa; IM: intestinal malabsorption; HO: hematopathy; RTA: renal tubular acidosis; JE: Japanese encephalitis; PA: pernicious anemia; AIH: autoimmune hepatitis; AS: ankylosing spondylitis; SA: asplenia. #: Case 4 and Case 5, Case 16 and Case 17, and Case 19 and Case 20 were siblings, respectively. *: homozygous mutations. The parents of cases 7 and 12 were consanguineous marriages. Cases 1 to 13 were from our center, and cases 14 to 25 were from the reported literature. GenBank accession number of *AIRE*: NM_000383.

## Data Availability

All data generated or analyzed during this study are included in this published article [and its supplementary information files].

## Abbreviations

AD: Addison’s disease
AIRE: Autoimmune regulator
APS1: Autoimmune polyendocrine syndrome type 1
AS: Ankylosing spondylitis
CMC: Chronic mucocutaneous candidiasis
HP: Hypoparathyroidism
RTA: Renal tubular acidosis
