# Supplementary material for: Characterization of the clinical and genetic spectrum of autoimmune polyendocrine syndrome type 1 in Chinese case series
Source: Orphanet J Rare Dis. 2021 Jul 3;16:296. doi: 10.1186/s13023-021-01933-y (PMC8254246; doi:10.1186/s13023-021-01933-y)
Supplement: Supplementary file 1 — Additional file 1. Table 1. Prevalence of Different Components in Chinese Patients with APS1. Abbreviation: APS1: Autoimmune Polyendocrine Syndrome Type 1. Table 2. Mutations of the AIRE Gene in Chinese Patients with APS1. Abbreviation: APS1: Autoimmune Polyendocrine Syndrome Type 1. GenBank accession number of AIRE: NM_000383. HSR: Homogeneously Staining Region; CARD: Caspase Recruitment Domain; NLS: Nuclear Localization Signal; PHD: Plant Homeodomain; Het: Heterozygous; Hom: Homozygous; “-” indicates that data are not available. CARD/HSR, amino acids 1–105; SAND: amino acids 181–280; two plant homeodomain (PHD) fingers type zinc fingers (amino acids 296–343 and 434–475); four LXXLL domains that are found on coactivators of nuclear receptors (amino acids 7–11, 63–67, 414–418, and 516–520) and a nuclear localization signal (amino acids 100–189). Figure 1. Clinical Spectrum and Genotype in Patients with APS1 in China. Abbreviation: APS1: autoimmune polyendocrine syndrome type 1; HP: hypoparathyroidism; CMC: chronic mucocutaneous candidiasis; AD: Addison’s disease; HT: hypothyroidism; HG: hypergonadotropic hypogonadism; T1DM: type 1 diabetes mellitus; ED: ectodermal dysplasia, including enamel dysplasia and nail dystrophy; A: alopecia; K: keratitis; RP: retinitis pigmentosa; IM: intestinal malabsorption; HO: hematopathy; RTA: renal tubular acidosis; JE: Japanese encephalitis; PA: pernicious anemia; AIH: autoimmune hepatitis; AS: ankylosing spondylitis; SA: asplenia. #: Case 4 and Case 5, Case 16 and Case 17, and Case 19 and Case 20 were siblings, respectively. *: homozygous mutations. The parents of cases 7 and 12 were consanguineous marriages. Cases 1 to 13 were from our center, and cases 14 to 25 were from the reported literature. GenBank accession number of AIRE: NM_000383. [file 13023_2021_1933_MOESM1_ESM.docx]

Supplemental Table 1. Prevalence of Different Components in Chinese Patients with APS1 (n=25)

| **Components** | **Prevalence (%)** |
| --- | --- |
| **Classic triad** |  |
| Hypoparathyroidism | 23 (92) |
| Chronic mucocutaneous candidiasis | 19 (76) |
| Addison’s disease | 18 (72) |
| Any of two | 11 (44) |
| All three | 12 (48) |
| **Other endocrinopathies** |  |
| Hypothyroidism | 9 (36) |
| Primary amenorrhea | 2 (8) |
| Diabetes mellitus type 1 | 2 (8) |
| **Ectodermal dysplasia** |  |
| Enamel dysplasia and nail dystrophy | 7 (28) |
| Hair loss | 10 (40%) |
| **Ocular manifestations** |  |
| Keratitis | 4 (16) |
| Retinitis pigmentation | 2 (8) |
| **Hematological diseases** |  |
| Myeloproliferative disease | 1 (4) |
| Pure red cell aplasia | 1 (4) |
| **Other less common complications** |  |
| Intestinal malabsorption | 3 (12) |
| Japanese encephalitis | 2 (8) |
| Pernicious anemia | 1 (4) |
| Renal tubular acidosis | 1 (4) |
| Asplenia | 1 (4) |
| Autoimmune hepatitis | 1 (4) |
| Ankylosing spondylitis | 1 (4) |

**Abbreviation:** APS1: Autoimmune Polyendocrine Syndrome Type 1.

**Supplemental Table 2. Mutations of the *AIRE* Gene in Chinese Patients with APS1.**

| Domain | Cases | Location in *AIRE* | Nucleotide alteration | Amino acids change | Heterozygosity | Reported outside China |
| --- | --- | --- | --- | --- | --- | --- |
| HSR/CARD | 4 | Exon 1 | c.T38C | p.L13P | Het | Yes |
|  | 14 | Exon 1 | c.47C>G | p.T16R | Het | No |
|  | 22 | Exon 1 | c.55G>A | p.A19T | Het | No |
|  | 7 | Exon 2 | c.A206C | p.Q69P | Hom | No |
|  | 2 | Exon 2 | c.A269G | p.Y90C | Hom | yes |
| NLS | 16 | Exon 3 | c.463G>A | p.G155S | Hom | No |
|  | 1 and 15 | Exon 4 | c.484dupC | p.K161fs | Hom | No |
| SAND | 24 | Exon 5 | c.622G>T | p.G208W | Het | No |
|  | 8 | Exon 5 | c.623G>T | p.G208V | Het | No |
|  | 22 | Exon 6 | c.769C>T | p.R257X | Het | Yes |
|  | 6 | Exon 6 | c.737delC | p.A246fs | Het | No |
| PHD1 | 6 | Exon 8 | c.C922T | p.L308F | Het | No |
| Entire gene | 25 | - | - | - | Het | Yes |
| Intron | 25 | IVS11 | +1G>A | - | Het | No |

**Abbreviation:** APS1: Autoimmune Polyendocrine Syndrome Type 1. GenBank accession number of AIRE: NM_000383. HSR: Homogeneously Staining Region; CARD: Caspase Recruitment Domain; NLS: Nuclear Localization Signal; PHD: Plant Homeodomain; Het: Heterozygous; Hom: Homozygous; “-” indicates that data are not available.

CARD/HSR, amino acids 1–105; SAND: amino acids 181–280; two plant homeodomain (PHD) fingers type zinc fingers (amino acids 296–343 and 434–475); four LXXLL domains that are found on coactivators of nuclear receptors (amino acids 7–11, 63–67, 414–418, and 516–520) and a nuclear localization signal (amino acids 100–189).

**Supplemental figure 1. Clinical spectrum and genotype in each patient with APS1 in China**.


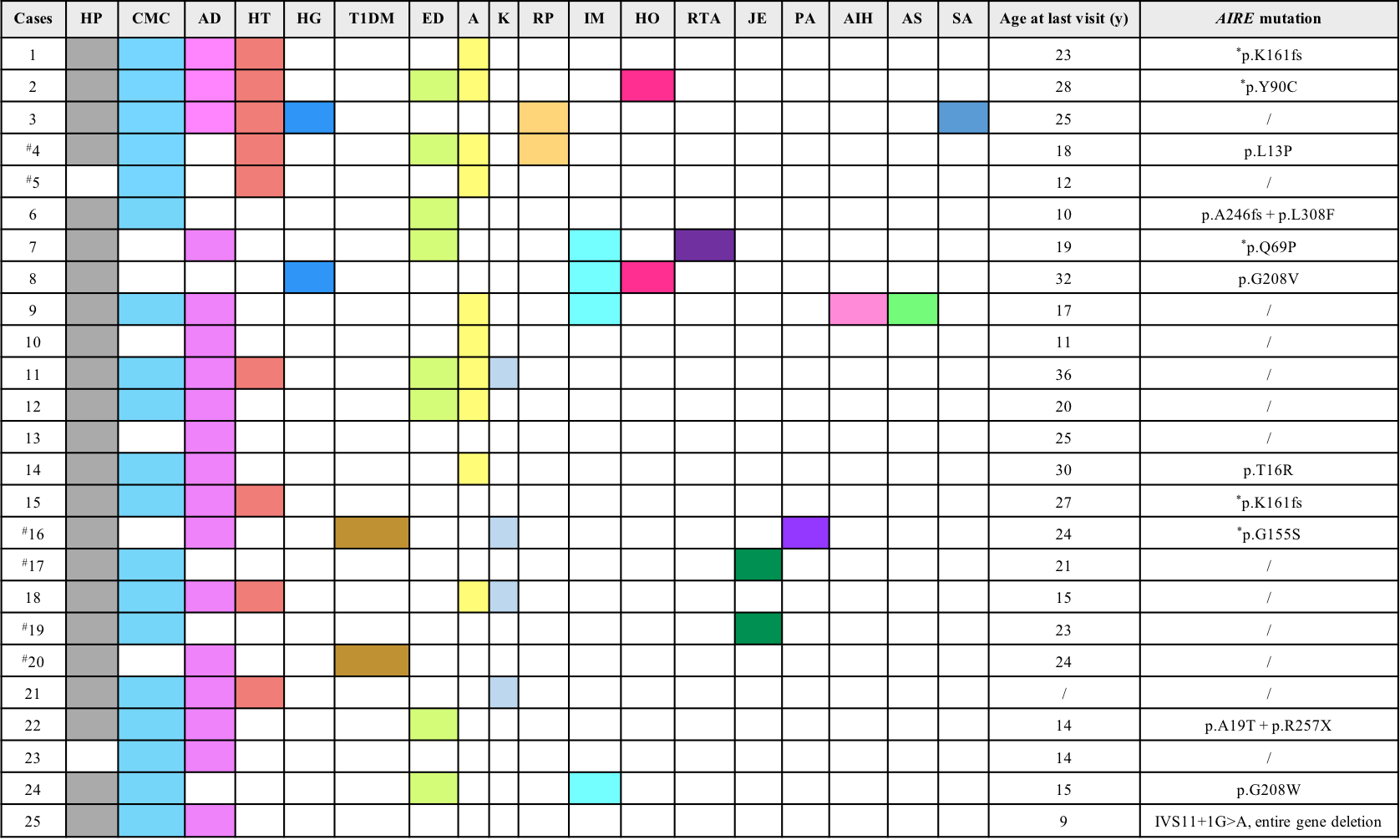


**Abbreviation:** APS1: autoimmune polyendocrine syndrome type 1; HP: hypoparathyroidism; CMC: chronic mucocutaneous candidiasis; AD: Addison’s disease; HT: hypothyroidism; HG: hypergonadotropic hypogonadism; T1DM: type 1 diabetes mellitus; ED: ectodermal dysplasias, including enamel dysplasia and nail dystrophy; A: alopecia; K: keratitis; RP: retinitis pigmentosa; IM: intestinal malabsorption; HO: hematopathy; RTA: renal tubular acidosis; JE: Japanese encephalitis; PA: pernicious anemia; AIH: autoimmune hepatitis; AS: ankylosing spondylitis; SA: asplenia.

#: Case 4 and case 5, case 16 and case 17, case 19 and case 20 were siblings, respectively. *: homozygous mutations. The parents of cases 7 and 12 were consanguineous marriages. Cases 1 to 13 were from our center, and cases 14 to 25 were from reported literature. GenBank accession number of *AIRE*: NM_000383.
